# Hypereosinophilia of persistent/unknown cause: a bone marrow–based study integrating WHO-HAEM5 criteria and myeloid next-generation sequencing

**DOI:** 10.1093/ajcp/aqag031

**Published:** 2026-05-10

**Authors:** Mohamed M Eletrebi, Juan Sanchez-Ramirez, Abdalla Abdalla, Carol J Holman

**Affiliations:** Department of Pathology, Roy J. and Lucille A. Carver College of Medicine, University of Iowa, Iowa City, IA, United States; Department of Pathology, Roy J. and Lucille A. Carver College of Medicine, University of Iowa, Iowa City, IA, United States; Department of Pathology, Roy J. and Lucille A. Carver College of Medicine, University of Iowa, Iowa City, IA, United States; Department of Pathology, Roy J. and Lucille A. Carver College of Medicine, University of Iowa, Iowa City, IA, United States

**Keywords:** chronic eosinophilic leukemia, clonal hematopoiesis, myeloid neoplasms, hypereosinophilic syndrome, hypereosinophilia, next-generation sequencing

## Abstract

**Objectives:**

Hypereosinophilia (HE), defined as an absolute eosinophil count (AEC) ≥1.5 × 10^9^/L, poses diagnostic challenges when distinguishing clonal myeloid neoplasms from idiopathic HE/hypereosinophilic syndrome, particularly under WHO-HAEM5 criteria. We characterized patients with HE of persistent/unknown cause (HEPU) undergoing bone marrow (BM) evaluation to refine diagnostic classification.

**Methods:**

We identified 307 BM biopsy specimens (2015-2023) mentioning “eosinophilia.” Patients with defined reactive or clonal etiologies were classified as group 2 (known etiology, n = 269). The remaining patients constituted group 1 (HEPU, n = 38). We reviewed clinical data, morphology, cytogenetics/fluorescence in situ hybridization (FISH), and myeloid next-generation sequencing (NGS), assigning final diagnoses via the fifth edition of the World Health Organization Classification of Haematolymphoid Tumours (WHO-HAEM5) criteria and the 2022 International Consensus Classification (ICC).

**Results:**

Compared to group 2, patients with HEPU were younger (median 48 vs 62 years; *P* = .004) and exhibited higher initial AECs (2.8 vs 0.6 × 10^9^/L; *P* < .001). Comprehensive workup reclassified the HEPU cohort (n = 38) into the idiopathic spectrum (36.8%), reactive eosinophilia (28.9%), clonal disorders including myeloid and lymphoid neoplasms (28.9%), and mixed reactive vs idiopathic (5.3%) categories. While cytogenetics/FISH were largely noncontributory (detecting *FIP1L1::PDGFRA* in 2 cases), myeloid NGS detected mutations in 47% (7/15) of tested patients with HEPU, involving genes such as *TP53*, *BCOR*, *SF3B1*, and *TET2*.

**Conclusions:**

Integrated clinicopathologic and molecular assessment reclassified most HEPU cases as reactive or clonal, with only 39% remaining idiopathic. Myeloid NGS frequently identifies clonal variants critical for refining HE classification, although results require cautious interpretation within the WHO-HAEM5 and ICC 2022 context.

Key Points

- Persistent unexplained hypereosinophilia is uncommon but often becomes reclassified as reactive or clonal after integrated bone marrow evaluation.
- Myeloid next-generation sequencing identifies clonal variants in nearly half of tested cases of persistent unexplained hypereosinophilia, and it reveals myeloid neoplasms as defined by the International Consensus Classification 2022 and the World Health Organization’s fifth edition in a subset.
- Distinguishing chronic eosinophilic leukemia from idiopathic hypereosinophilic syndrome in both classifications requires combined assessment of bone marrow morphology, cytogenetics/fluorescence in situ hybridization, and next-generation sequencing.

## INTRODUCTION

Hypereosinophilia (HE) is typically defined as a persistent absolute eosinophil count ≥1.5 × 10^9^/L; when accompanied by eosinophil-mediated tissue or organ injury, it is termed hypereosinophilic syndrome (HES).^1^^,^^2^ Because HE spans secondary/reactive conditions and primary/clonal hematologic disorders, determining etiology is central to risk stratification and management, including identifying patients with targetable lesions.^3-5^ Importantly, HE can also accompany lymphoid neoplasms, including B- or T-cell acute lymphoblastic leukemia/lymphoblastic lymphoma, and should remain in the differential when eosinophilia is being evaluated by bone marrow (BM) biopsy. A well-recognized example is B-cell acute lymphoblastic leukemia/lymphoblastic lymphoma with t(5; 14)(q31; q32)/IGH::IL3, in which IL-3 overexpression drives marked peripheral blood and marrow eosinophilia; T-cell lymphoblastic lymphoma with eosinophilia has also been described, sometimes in syndromic association with subsequent myeloid neoplasia.^6^^,^^7^

Current diagnostic practice is framed by 2 contemporary classification systems: fifth edition of the World Health Organization Classification of Haematolymphoid Tumours (WHO-HAEM5) and the 2022 International Consensus Classification (ICC). Both recognize myeloid/lymphoid neoplasms with eosinophilia driven by tyrosine kinase (MLN-eo-TK) gene fusions as a distinct, often highly actionable group.^5^^,^^8-10^ For patients without defining fusions, the major challenge is separating idiopathic HE/HES (including HE of undetermined significance) from chronic eosinophilic leukemia (CEL). The ICC places stronger emphasis on BM morphologic evidence of a myeloid neoplasm in this distinction, consistent with data showing marrow morphology is a strong discriminator between CEL not otherwise specified (NOS) and reactive/idiopathic HE/HES.^8^^,^^9^^,^^11^

Broader use of myeloid next-generation sequencing (NGS) adds diagnostic resolution in selected cases but also introduces interpretive pitfalls, as variants associated with age-related clonal hematopoiesis of indeterminate potential (CHIP) can be detected in the absence of overt neoplasia and may not be the driver of eosinophilia.^12-15^ In this retrospective BM-based study, we evaluated patients with persistent and unexplained HE (HEPU) at the time of biopsy to determine how often integrated clinicopathologic assessment (morphology, cytogenetics/fluorescence in situ hybridization [FISH], and molecular testing) reclassifies these cases and to describe the diagnostic yield and context for interpreting myeloid NGS findings under WHO-HAEM5 and ICC 2022 frameworks.^9^^,^^16^

## MATERIALS AND METHODS

### Study design and case selection

We performed a retrospective cohort study at the University of Iowa Health Care, approved by the institutional review board, and informed consent was waived due to minimal risk and use of deidentified data. The laboratory information system was queried for all BM biopsies reported between January 2015 and December 2023 that contained the term *eosinophilia*. Peripheral blood absolute eosinophil counts (AECs) at or near the time of biopsy were recorded.

We identified 307 patients with HE and adequate BM material, which constituted the study cohort.

Patients were divided into 2 groups based on the status at the time of the BM biopsy. Group 2 (known-etiology eosinophilia) comprised 269 patients with a defined underlying cause of HE at biopsy (eg, previously diagnosed WHO-defined myeloid/lymphoid neoplasm, solid tumor, parasitic infection, allergic/atopic disease, autoimmune disease, or drug reaction). Group 1 (HEPU) comprised 38 patients in whom no reactive, clonal, or familial etiology was identified at the time of BM evaluation.

### Clinical data and assessment of organ involvement

Electronic medical records were reviewed for demographics, indications for BM biopsy, AECs (initial and maximal documented values), other hematologic parameters, and clinical evidence of organ involvement. Organ damage was recorded when clearly documented by ­treating clinicians based on symptoms, imaging, or tissue biopsy (eg, endomyocardial involvement, eosinophilic pneumonia, gastroenteritis, dermatitis, or neurologic dysfunction) and attributed to HE by the clinical team.

### Definitions and diagnostic criteria

Diagnostic terminology and final diagnostic assignment followed the 2022 WHO-HAEM5 and were additionally mapped to the 2022 ICC. Idiopathic HES was defined as HE with organ damage and no identifiable reactive or clonal cause. Idiopathic HE without organ damage was termed hypereosinophilia of undetermined significance (HEUS). Cases with overlapping features (eg, HE with borderline organ findings or limited follow-up) were designated “reactive vs idiopathic HES” and considered part of the idiopathic spectrum in aggregate analyses. Borderline organ findings were defined as clinical symptoms (eg, mild dyspnea, nonspecific abdominal pain) or imaging abnormalities (eg, transient pulmonary opacities, borderline cardiac wall thickening) that were suggestive of eosinophil-mediated injury but lacked definitive histologic confirmation or failed to meet the full consensus diagnostic criteria for established HES-associated organ damage at the time of evaluation.

Chronic eosinophilic leukemia and other clonal myeloid neoplasms with eosinophilia were diagnosed when there was (1) morphologic evidence of a myeloid neoplasm involving the BM and/or blood, (2) exclusion of *BCR::ABL1*-positive chronic myeloid leukemia and classic myeloproliferative neoplasms, and (3) demonstration of clonality by cytogenetic or molecular genetic testing (recurrent rearrangements or somatic mutations), interpreted in the context of age and coexisting hematologic features.

### Cytogenetic and FISH studies

Conventional karyotyping was performed on BM aspirates using standard G-banded metaphase analysis in a Clinical Laboratory Improvement Amendments–certified laboratory. At least 20 metaphases were analyzed whenever possible. Abnormalities were reported according to the International System for Human Cytogenomic Nomenclature.

Fluorescence in situ hybridization panels were performed and included probes for *FIP1L1*::*PDGFRA*, *PDGFRB*, *FGFR1*, *PCM1-JAK2*, and other rearrangements relevant to myeloid neoplasms with eosinophilia. Additional FISH probes (eg, for recurrent myeloid abnormalities such as t(8;21), inv(16), t(15;17), and *TP53* deletions) were used when clinically indicated. Results were interpreted using laboratory-validated cutoffs.

### Next-generation sequencing

In a subset of 9 patients with HEPU, myeloid NGS was performed on DNA extracted from archival formalin-fixed, paraffin-embedded BM biopsy specimens using the acute myeloid leukemia mutation profiling assay. This semiconductor-based targeted panel interrogates hotspot or coding regions of 35 myeloid genes (including *ASXL1*, *BCOR*, *CEBPA*, *DNMT3A*, *FLT3*, *IDH1*, *IDH2*, *JAK2*, *KIT*, *NPM1*, *RUNX1*, *SF3B1*, *SRSF2*, *TET2*, *TP53*, and others) and detects single-nucleotide variants and small insertions/deletions (≤50 bp). Libraries were prepared as barcoded amplicon pools from formalin-fixed, paraffin-embedded derived DNA, sequenced, and aligned to the hg19 reference genome using a laboratory-validated pipeline. Variants were annotated with GenomOncology software, and nonsynonymous exonic variants, splice site alterations, and small indels with population minor allele frequency >0.005 were reported. Detected variants were classified as pathogenic/likely pathogenic or variants of uncertain significance according to the Association for Molecular Pathology, American Society of Clinical Oncology, and College of American Pathologists guidelines and interpreted in the context of patient age, cytopenias, and BM morphology.

### Assignment of final diagnoses

For HEPU cases, all clinical, morphologic, cytogenetic, FISH, and molecular data were integrated and final diagnoses assigned by consensus among the hematopathologists, using WHO-HAEM5 criteria. Cases were categorized at the end of follow-up as (1) reactive eosinophilia, (2) idiopathic spectrum HE/HES (HEUS, idiopathic HES, idiopathic HES vs CEL/reactive vs idiopathic), (3) clonal myeloid eosinophilia (MLN-eo-TK or other WHO-defined myeloid neoplasm with eosinophilia), or (4) lymphoid HES.

### Statistical analysis

Continuous variables were summarized as medians with interquartile ranges and compared between groups using the Mann-Whitney *U* test. Categorical variables were compared using the χ^2^ test or Fisher exact test, as appropriate. A 2-sided *P* value <.05 was considered statistically significant. All analyses were performed using jamovi statistical software (version 2.6.44; The jamovi project).

## RESULTS

### Study cohort and group assignment

**Figure 1:**
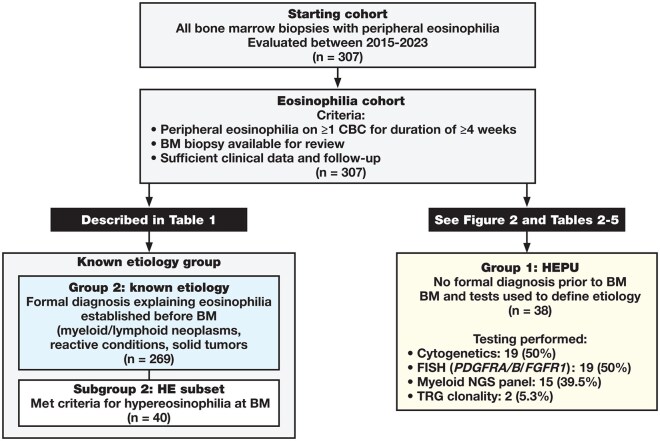
Case selection and grouping of patients with eosinophilia. Flow diagram depicting selection of patients for the study. All bone marrow biopsy specimens with peripheral eosinophilia evaluated between 2015 and 2023 were screened, and cases with adequate clinical data and interpretable bone marrow specimens were included in the eosinophilia cohort (n = 307). Patients were then divided into group 2, comprising those with eosinophilia of previously known etiology at the time of bone marrow biopsy (n = 269), and group 1, comprising patients with hypereosinophilia of persistent/unknown cause at biopsy (HEPU; n = 38). Group 2 is characterized in Table 1, whereas group 1 forms the primary focus of the present study and is further detailed in Figure 2 and Tables 1 to 5.

A total of 269 cases with eosinophilia of previously known etiology (group 2) and 38 cases with persistent, unexplained HE at the time of BM biopsy (group 1, HEPU) were included in the final analysis (Figure 1).

Cases were assigned to group 2 when a definitive underlying diagnosis associated with eosinophilia had been established prior to the index marrow and to group 1 when no formal diagnosis was present, and the BM evaluation formed the basis for etiologic classification.

### Eosinophilia of previously known etiology (group 2)

Among the 269 patients with eosinophilia of previously known etiology (group 2), myeloid and lymphoid neoplasms accounted for most cases, with myeloid neoplasms representing approximately one-third of the cohort (Table 1). Solid tumors, reactive conditions, and other hematologic diagnoses comprised the remainder. Overall, patients in group 2 were older (median age 62 years; IQR, 49-70 years), with a modest male predominance.

**Table 1:** Demographic and Clinical Characteristics of Patients With Eosinophilia of Previously Known Etiology (Group 2, n = 269)

Main diagnosis	No. of cases	Male	Female	M/F ratio	Age range, y	Age, mean (IQR), y	AEC range/mm³	Median AEC/mm³	AEC IQR/mm³	% of total cases
Myeloid neoplasms	94	53	40	—	—	—	—	—	—	34.7
Myeloproliferative neoplasm	32	17	14	1.2	0-85	51 (36-68)	90-7552	1550	715-4530	11.6
Myelodysplastic neoplasm	12	6	6	1.0	22-88	70 (68-77)	150-3440	645	402-910	4.5
MDS/MPN	9	6	3	2.0	54-79	70 (69-72)	80-6590	926	355-1383	3.4
Acute myeloid leukemia	38	21	17	1.2	17-89	52 (38-67)	0-8500	922	634-1401	14.2
MLN-eo-TK	2	2	0	—	29-40	35 (32-37)	1780-4490	2690	1790-3590	0.7
MPAL	1	1	0	—	66	66	1040	1040	—	0.4
Lymphoid neoplasms	147	92	55	—	—	—	—	—	—	54.9
B-cell lymphoblastic leukemia	10	7	3	2.3	22-88	42 (35-45)	400-6682	925	676-1112	3.7
CLL/SLL	4	3	1	3.0	50-65	60 (57-64)	560-1630	638	433-944	1.5
DLBCL	8	5	3	1.7	28-85	58 (48-67)	110-12 977	925	760-1726	3.0
Hodgkin lymphoma	4	3	1	3.0	21-61	34 (25-37)	320-1460	698	550-923	1.5
Plasma cell neoplasms	108	66	42	1.6	23-88	65 (60-70)	110-5090	710	555-1162	40.3
Other lymphoid neoplasms	13	8	5	1.6	38-76	63 (61-72)	21-2020	750	645-865	4.9
Solid neoplasm	9	4	5	0.8	0-77	27 (0-58)	315-6510	858	669-1098	3.4
Other conditions	19	9	10	—	—	—	—	—	—	7.1
Reactive eosinophilia	9	4	5	0.8	3-73	48 (33-60)	440-2390	850	620-1450	3.4
Idiopathic eosinophilia	5	2	3	0.7	31-76	55 (47-64)	420-1720	970	660-1145	1.9
Neutropenia	3	2	1	2.0	7-49	34 (26-47)	410-830	545	528-655	1.1
Aplastic anemia	2	1	1	1.0	36-55	46 (41-50)	733-1720	1147	940-1353	0.7

Abbreviations: AEC, absolute eosinophil count; CLL/SLL, chronic lymphocytic leukemia/small lymphocytic lymphoma; DLBCL, diffuse large B-cell lymphoma; MDS, myelodysplastic neoplasm; MLN-eo-TK, myeloid/lymphoid neoplasms with eosinophilia and tyrosine kinase gene fusions; MPAL, mixed-phenotype acute leukemia; MPN, myeloproliferative neoplasm.

### Patients with persistent unexplained HE at biopsy (group 1, HEPU) baseline characteristics

The HEPU group (group 1) comprised 38 patients without a formal underlying diagnosis at the time of BM biopsy. The median age at biopsy was 48 years (IQR, 33-65 years), with a range of 4 to 89 years, and 22 of 38 (57.9%) were female. The median initial AEC among patients with available data (n = 34) was 2795 cells/mm³ (IQR, 1178-4425; range, 0-23 645), and the median documented peak AEC was 3833 cells/mm³ (IQR, 2848-9823; range, 1500-81 800). Overall, 11 of 38 (28.9%) patients had documented eosinophil-associated organ involvement at the time of evaluation, most commonly affecting the lungs (5/38, 13.2%), skin (3/38, 7.9%), and gastrointestinal tract, including eosinophilic gastroenteritis or esophagitis (3/38, 7.9%), with less frequent cardiac (2/38, 5.3%) and musculoskeletal/soft tissue (eosinophilic fasciitis; 1/38, 2.6%) manifestations. The remaining 27 of 38 (71.1%) had no clear evidence of eosinophil-mediated organ damage documented at baseline. Conventional cytogenetic analysis was performed in 19 of 38 (50.0%) patients with HEPU, and all 19 demonstrated a normal karyotype. Fluorescence in situ hybridization studies targeting *PDGFRA/PDGFRB/FGFR1* and related myeloid loci (including *BCR::ABL1* [to exclude chronic myeloid leukemia], *PML::RARA*, *RUNX1::RUNX1T1*, *CBFB::MYH11*, *KMT2A* [*MLL*], *MECOM* [*EVI1*], and *TP53* deletions, as clinically indicated) were also performed in 19 of 38 (50.0%) patients and were normal/negative in 16 of 19 (84.2%), indeterminate in 1 of 19 (5.3%), and positive for a *FIP1L1*::*PDGFRA* fusion in 2 of 19 (10.5%) cases (Table 2).

**Table 2:** Baseline Characteristics of Patients With Persistent and Unexplained Hypereosinophilia at Biopsy (Group 1, HEPU; n = 38)^a^

Variable	Group 1 (HEPU, n = 38)
Sex, No. (%)	
Male	16 (42.1)
Female	22 (57.9)
Age, median (IQR), y	48 (33-65)
Eosinophil counts	
Initial AEC,^a^ cells/mm³, median (IQR)	2795 (1178-4425)
Initial AEC,^a^ cells/mm³, range	0-23 645
Maximal AEC, cells/mm³, median (IQR)	3833 (2848-9823)
Maximal AEC, cells/mm³, range	1500-81 800
Missing initial AEC, No. (%)	4 (10.5)
Organ involvement, No. (%)	
Any documented organ involvement	11 (28.9)
Skin involvement	3 (7.9)
Gastrointestinal involvement	3 (7.9)
Esophageal involvement	2 (5.3)
Pulmonary involvement	5 (13.2)
Cardiac involvement	2 (5.3)
Musculoskeletal/soft tissue involvement	1 (2.6)
Testing performed	
Conventional cytogenetics performed, No. (%)	19 (50.0)
Normal karyotype, No./total No. tested (%)	19/19 (100)
FISH (*PDGFRA/PDGFRB/FGFR1* or myeloid panel) performed, No. (%)	19 (50.0)
FIP1L1-PDGFRA fusion detected, No./total No. tested (%)	2/19 (10.5)
Indeterminate FISH result, No./total No. tested (%)	1/19 (5.3)
Normal/negative FISH, No./total No. tested (%)	16/19 (84.2)
Myeloid NGS/molecular panel performed, No. (%)	15 (39.5)
≥1 mutation detected on myeloid panel, No./total No. tested (%)	7/15 (46.7)
No mutation detected, No./total No. tested (%)	7/15 (46.7)
Insufficient material for NGS, No./total No. tested (%)	1/15 (6.7)
TRG clonality assay performed, No. (%)	2 (5.3)
Clonal/oligoclonal TRG rearrangement, No./total No. tested (%)	2/2 (100)

Abbreviations: AEC, absolute eosinophil count; FISH, fluorescence in situ hybridization; HEPU, hypereosinophilia of persistent/unknown cause; NGS, next-generation sequencing.

a Initial AEC statistics calculated for patients with nonmissing values (n = 34).

### Molecular testing and NGS findings in HEPU

Beyond cytogenetics and FISH, myeloid NGS was available in a subset of 15 of 38 (39.5%) patients with HEPU, including 6 cases evaluated at the time of diagnosis and 9 cases tested retrospectively as part of this research study. This limited number is in part due to physician discretion and the availability of tissue (some cases are from outside institutions). Across all 15 patients, 7 of 15 (46.7%) harbored at least 1 variant (somatic, germline, or variant of uncertain clinical significance [VUS]), 7 of 15 (46.7%) had no reportable variants, and 1 of 15 (6.7%) had a noninformative study due to insufficient DNA. In the clinical cohort (6 cases), 4 panels were negative, and 2 showed reportable alterations: 1 patient carried *RUNX1* c.167T > C (p.L56S) and *TET2* c.4715G > A (p.R1572Q) variants interpreted as of uncertain clinical significance, and another had a reported *TET2* Q1529fs (c.4585delC55.78).

The research acute myeloid leukemia mutation panel was performed in 9 patients with HEPU, with mutations detected in 5 of 9 (55.6%), no variants in 3 of 9 (33.3%), and 1 failed assay (11.1%) (Table 3). Detected variants involved canonical myeloid and CHIP-associated genes, including *CEBPA* S251N (c.752G > A; variant allele frequency [VAF] 5.6%) in 1 case; a combination of *BCOR* W1218* (c.3653G > A; VAF 2.95%), *CBL* Y371H (c.1111T > C; VAF 48.4%), and *SF3B1* K666N (c.1998G > T; VAF 45.0%) in another; *BCOR* c.4977-4delGinsTT (VAF 43.7%) in a third case; and 2 distinct *TP53* variants, p.Arg248Gln (c.743G > A; VAF 21%) and Y220C (c.659A > G; VAF 2.2%), in 2 separate patients. Together, these findings show that, in a subset of patients with HEPU, targeted NGS can uncover clonal myeloid-associated variants, including *TP53* and epigenetic/spliceosome genes, even when cytogenetics is normal and FISH is unrevealing.

**Table 3:** Research AML Mutation Panel Results in Patients With Persistent and Unexplained Hypereosinophilia (Group 1, HEPU; n = 9)^a^

Case No.	Result category	Gene	Protein change	cDNA change	VAF, %	Reads	Chr: Position	Classification in report
1	No mutation detected	—	—	—	—	—	—	No reportable variants detected on study AML panel
2	Mutation detected	*CEBPA*	S251N	c.752G > A	5.56	22	19:33792569	Variant of uncertain clinical significance
3	Mutations detected	*BCOR*	W1218*	c.3653G > A	2.95	58	X:39923055	Variant with potential clinical significance
3	Mutations detected	*CBL*	Y371H	c.1111T > C	48.43	631	11:119148891	Variant with potential clinical significance
3	Mutations detected	*SF3B1*	K666N	c.1998G > T	44.97	1797	2:198267359	Variant with potential clinical significance
4	Insufficient material	—	—	—	—	—	—	NGS not interpretable due to insufficient DNA
5	No mutation detected	—	—	—	—	—	—	No reportable variants detected on study AML panel
6	Mutation detected	*TP53*	p.Arg248Gln	c.743G > A	21	NA	19:7577538	Variant with potential clinical significance
7	No mutation detected	—	—	—	—	—	—	No reportable variants detected on study AML panel
8	Mutation detected	*BCOR*	Not specified	c.4977-4delGinsTT	43.71	299	X:3991657	Variant of uncertain clinical significance
9	Mutation detected	*TP53*	Y220C	c.659A > G	2.2	3999	17:7578190	Variant with potential clinical significance

Abbreviations: AML, acute myeloid leukemia; cDNA, complementary DNA; HEPU, hypereosinophilia of persistent/unknown cause; NA, **not available**; NGS, next-generation sequencing; VAF, variant allele frequency.

a Please note that this sequence of cases is not the same as the exemplary cases discussed in the Results section.

### Comparison between HEPU (group 1) and eosinophilia of known etiology (group 2)

When compared with patients whose eosinophilia had a previously established etiology (group 2, n = 269), patients with HEPU (group 1) were significantly younger (median age 48 [IQR, 33-65] vs 62 [IQR, 49-70] years; *P* = .004). Regarding sex distribution, group 2 exhibited a male predominance (58.7% male) in contrast to the female predominance observed in group 1 (42.1% male). While this difference showed a notable trend, it did not reach statistical significance in this cohort (*P* = .08). Peak eosinophil counts were markedly higher in HEPU than in group 2 (median maximal AEC 3833 [IQR, 2848-9823] vs 1005 [IQR, 719-1730] cells/mm³; *P* < .001), whereas initial AEC values also tended to be higher in HEPU (median 2795 vs 600 cells/mm³). A substantial proportion of group 2 patients carried a defined myeloid or lymphoid malignancy or solid tumor diagnosis at baseline, and in these cases, eosinophilia was attributed to the primary disease process. In contrast, group 1 was enriched for patients requiring extensive diagnostic workup, including more frequent use of marrow-based molecular testing and for whom the final classification hinged on subtle morphologic features, molecular findings, and longitudinal clinical follow-up (Table 4).

**Table 4:** Comparison of Selected Features Between Hypereosinophilia of Persistent/Unknown Cause (Group 1, HEPU) and Eosinophilia of Known Etiology (Group 2)^a^

Feature	Group 1 (HEPU, n = 38)	Group 2 (known etiology, n = 269)	*P* value
Demographics			
Age, median (IQR), y	48 (33-65)	62 (49-70)	.004
Male sex, No./total No. (%)	16/38 (42.1)	158/269 (58.7)	.078
Eosinophil counts			
Initial AEC, cells/mm³, median (IQR)	2795 (1178-4425)	600 (448-960)	<.001
Maximal AEC, cells/mm³, median (IQR)	3833 (2848-9823)	1005 (719-1730)	<.001

Abbreviations: AEC, absolute eosinophil count; HEPU, hypereosinophilia of persistent/unknown cause.

a Values are shown as median (IQR) for continuous variables and No./total No. (%) for categorical variables. *P* values for age and eosinophil counts are from Mann-Whitney *U* tests; *P* value for sex distribution is from χ^2^ test.

### Final diagnostic classification within HEPU (group 1)

**Figure 2:**
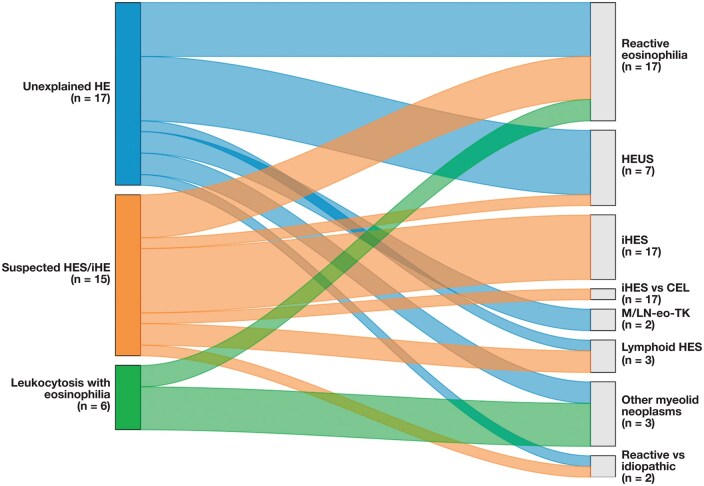
Flow of group 1 (hypereosinophilia of persistent/unknown cause [HEPU]) patients from pre–bone marrow working diagnosis to final integrated diagnosis. Sankey diagram shows the distribution of 38 patients with HEPU from pre–bone marrow working diagnosis categories (left) to final integrated diagnosis categories (right). Prebiopsy impressions were grouped as unexplained hypereosinophilia (HE; n = 17), suspected hypereosinophilic syndrome (HES)/idiopathic HE (n = 15), and leukocytosis with eosinophilia (n = 6). Final diagnoses included reactive eosinophilia (n = 11), hypereosinophilia of uncertain significance (HEUS; n = 7), idiopathic HES (n = 6), idiopathic HES vs chronic eosinophilic leukemia (CEL; n = 1), myeloid/lymphoid neoplasms with eosinophilia (M/LN-eo-TK) (n = 2), lymphoid HES (n = 3), other myeloid neoplasms (n = 6), and mixed “reactive vs idiopathic” categories (n = 2). Bandwidth is proportional to the number of patients, illustrating how bone marrow evaluation and ancillary testing reclassified a substantial fraction of patients from nonspecific prebiopsy labels to specific reactive, idiopathic, or clonal eosinophilic disorders.

At the time of BM biopsy, patients with HEPU were typically labeled with nonspecific working diagnoses such as “HE,” “HES,” or “leukocytosis with eosinophilia.” When grouped into 3 prebiopsy categories—unexplained HE (n = 17), suspected HES/idiopathic HE (n = 15), and leukocytosis with eosinophilia (n = 6)—subsequent integration of marrow morphology, cytogenetics, FISH, molecular testing, and clinical follow-up resulted in substantial reclassification (Table 5). Overall, 11 of 38 (28.9%) patients were ultimately diagnosed with reactive eosinophilia, 14 of 38 (36.8%) remained within the idiopathic spectrum (idiopathic HES, HEUS, or idiopathic HES vs CEL), 11 of 38 (28.9%) were reclassified as having clonal disorders (other myeloid neoplasms, MLN-eo-TK, or lymphoid HES), and 2 of 38 (5.3%) were left in mixed “reactive vs idiopathic” categories. Notably, leukocytosis with eosinophilia as the prebiopsy impression most often corresponded to an underlying myeloid neoplasm (4/6, 66.7%), whereas patients initially labeled as having suspected HES/idiopathic HE were more frequently assigned to idiopathic HES or HEUS after full workup. Transitions from pre-BM working diagnoses to final integrated categories are summarized in Table 5 and visualized in Figure 2.

**Table 5:** Change in Diagnostic Classification in Group 1 (HEPU): Pre–Bone Marrow Working Diagnosis vs Final Integrated Diagnosis (n = 38)

Pre–bone marrow working diagnosis^a^	Reactive eosinophilia, No.	HEUS, No.	Idiopathic HES, No.	Idiopathic HES vs CEL, No.	M/LN-eo-TK, No.	Lymphoid HES, No.	Other myeloid neoplasm, No.	Reactive vs idiopathic, No.	Total No.
Unexplained hypereosinophilia (n = 17)	5	6	0	0	2	1	2	1	17
Suspected HES/idiopathic HE (n = 15)	4	1	6	1	0	2	0	1	15
Leukocytosis with eosinophilia (n = 6)	2	0	0	0	0	0	4	0	6
Total	11	7	6	1	2	3	6	2	38

Abbreviations: AEC, absolute eosinophil count; CEL, chronic eosinophilic leukemia; HE, hypereosinophilia; HEPU, hypereosinophilia of persistent/unknown cause; HES, hypereosinophilic syndrome; HEUS, hypereosinophilia of uncertain significance; MLN-eo-TK, myeloid/lymphoid neoplasms with eosinophilia and tyrosine kinase gene fusions.

a Pre–bone marrow categories reflect the clinical working impression at the time of biopsy and were grouped as follows: “Unexplained hypereosinophilia” includes hypereosinophilia and pancytopenia with hypereosinophilia; “Suspected HES/idiopathic HE” includes HES, chronic eosinophilia (HES vs idiopathic HE [iHE]), HES and Alport syndrome, and asthma vs iHE; “Leukocytosis with eosinophilia” includes leukocytosis with hypereosinophilia and leukocytosis with eosinophilia.

### Exemplary cases

Case 1. Presentation: A 67-year-old woman with a 1-year history of persistent HE (AEC 12.04 × 10^9^/L) and mediastinal lymphadenopathy.

**Figure 3:**
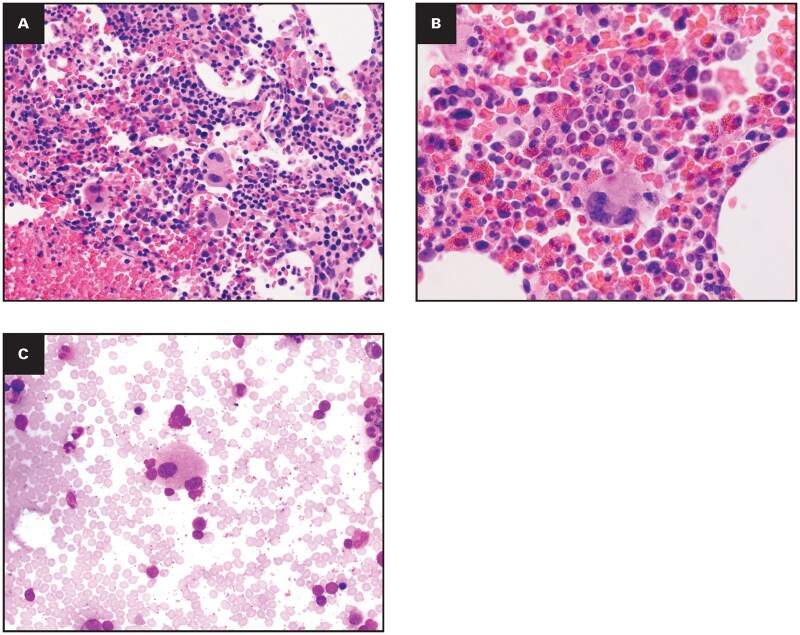
Exemplary case 1—borderline chronic eosinophilic leukemia vs idiopathic hypereosinophilic syndrome. (A) Bone marrow core biopsy specimen shows a moderately hypercellular marrow with trilineage hematopoiesis admixed with atypical megakaryocytes (hematoxylin and eosin [H&E], 20×). (B) Higher-power view highlights numerous mature eosinophils but without a clear increase in blasts (H&E, 50×). (C) Bone marrow aspirate smear demonstrates a dysplastic megakaryocyte with small and hypolobated nuclei (Wright-Giemsa, 100×).

Workup: BM showed 20% eosinophils and dysplastic megakaryocytes (small, hypolobated forms with focal clustering) (Figure 3). Karyotype and FISH (*PDGFRA/B*, *FGFR1*, *BCR-ABL1*) were negative. Myeloid NGS identified a p.Y220C *TP53* variant at a low VAF of 2.2%.

Final Diagnosis: Chronic eosinophilic leukemia (WHO-HAEM5)/CEL NOS (ICC 2022).

Diagnostic Lesson: This case illustrates the “diagnostic tension” in current guidelines. While the combination of persistent HE, megakaryocytic dysplasia, and a clonal mutation technically fulfills the criteria for CEL, the low VAF in an elderly patient blurs the line between overt leukemia and clonal hematopoiesis (CHIP).

Case 2. Presentation: A 68-year-old woman with secondary Sjögren syndrome and a 2-month history of eosinophilia (AEC 3.9 × 10^9^/L).

Workup: BM was hypercellular with increased eosinophils but lacked definitive dysplasia or blast increase. Next-generation sequencing identified a p.Arg248Gln *TP53* variant at 21% VAF. Notably, AEC normalized (0.3 × 10^9^/L) after a brief course of prednisone.

Final Diagnosis: Reactive/medication-related eosinophilia with coexisting *TP53*-mutant CHIP.

Diagnostic Lesson: This case demonstrates that even high-VAF pathogenic mutations can represent CHIP rather than malignancy if the eosinophilia is transient and marrow morphology remains nondiagnostic.

Case 3. Presentation: A 31-year-old woman with a 7-year history of chronic HE (AEC 1.7 × 10^9^/L), fatigue, and night sweats.

**Figure 4:**
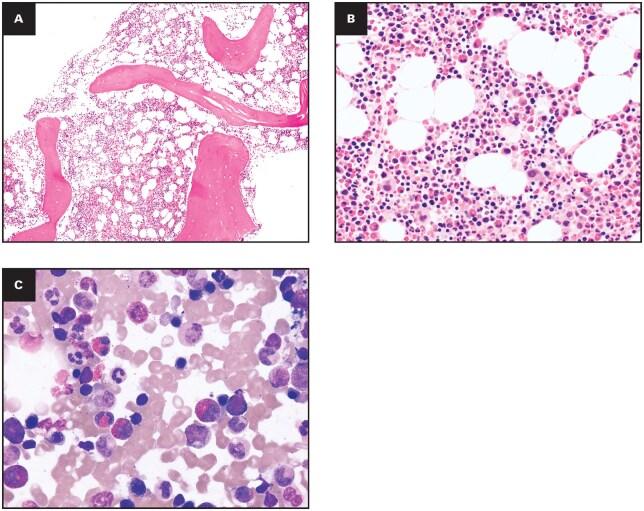
Exemplary case 3—idiopathic hypereosinophilic syndrome with *CEBPA* variant of uncertain significance. (A) Bone marrow core biopsy specimen at low power shows hypocellular marrow for age with preserved trilineage hematopoiesis and an interstitial increase in eosinophils (hematoxylin and eosin [H&E], 10×). (B) Higher-power view highlights numerous mature eosinophils without significant multilineage dysplasia or blast increase (H&E, 50×). (C) Bone marrow aspirate smear demonstrates persistent hypereosinophilia with morphologically mature eosinophils and otherwise orderly myeloid and erythroid maturation (Wright-Giemsa, 100×).

Workup: BM was hypocellular (30%-50%) with 16% eosinophils but no multilineage dysplasia (Figure 4). Karyotype and standard FISH were normal. Next-generation sequencing identified a p.S251N *CEBPA* variant (VAF 5.5%), classified as a VUS.

Final Diagnosis: Idiopathic HES (WHO-HAEM5/ICC 2022).

Diagnostic Lesson: In the absence of definitive marrow dysplasia or known pathogenic drivers, molecular VUS should not trigger a diagnosis of CEL/CEL NOS.

Case 4. Presentation: A 19-year-old man with long-standing gastrointestinal symptoms, suspected Crohn disease, and persistent HE (AEC 4.2 × 10^9^/L).

**Figure 5:**
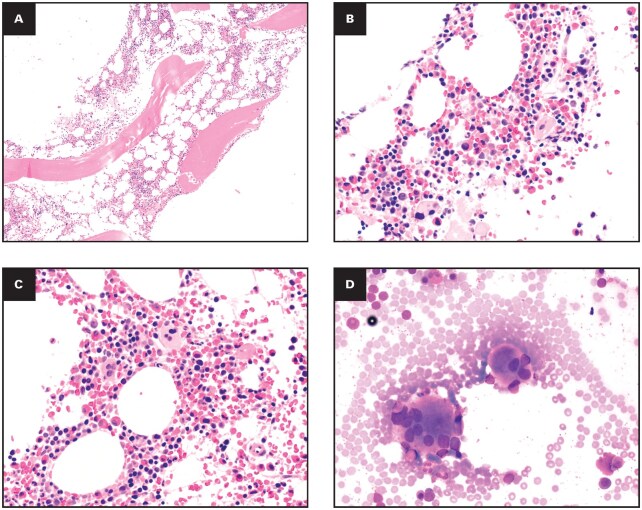
Exemplary case 4—idiopathic hypereosinophilic syndrome with dysplastic megakaryocytes and *BCOR* variant of uncertain significance. (A) Bone marrow core biopsy specimen at low power shows hypocellular marrow for age with relatively decreased trilineage hematopoiesis (hematoxylin and eosin [H&E], 10×). (B, C) Higher-power views highlight numerous mature eosinophils admixed with morphologically atypical megakaryocytes, including small hypolobated forms (H&E, 50×). (D) Bone marrow aspirate smear demonstrates dysplastic megakaryocytes with separated, irregularly lobated nuclei (Wright-Giemsa, 50×).

Workup: BM was hypocellular (40%) with increased eosinophils (26%) and dysplastic megakaryocytes (Figure 5). Next-generation sequencing identified a c.4977-4delGinsTT *BCOR* variant at a high VAF (43.71%), also classified as a VUS.

Final Diagnosis: Idiopathic HES.

Diagnostic Lesson: High-VAF variants do not automatically indicate malignancy if their pathogenic significance is unknown. This case underscores the conservative approach required when interpreting molecular data in the absence of established drivers.

Case 5. Presentation: A 67-year-old woman with multiple comorbidities presenting with leukocytosis (30 × 10^9^/L), marked HE (AEC 8 × 10^9^/L), anemia, and thrombocytosis.

**Figure 6:**
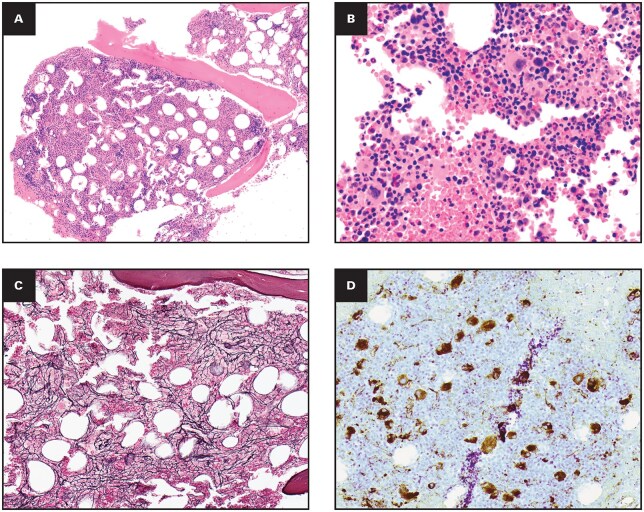
Exemplary case 5—myelodysplastic/myeloproliferative neoplasm with *SF3B1* mutation, thrombocytosis, and eosinophilia. (A) Bone marrow core biopsy specimen at low power shows a markedly hypercellular marrow for age with trilineage hematopoiesis and prominent megakaryocytic hyperplasia (hematoxylin and eosin [H&E], 10×). (B) Higher-power view highlights numerous small, hypolobated megakaryocytes in a background of increased eosinophils and left-shifted granulopoiesis (H&E, 50×). (C) Reticulin stain demonstrates increased reticulin myelofibrosis (MF 1-2). (D) Immunohistochemistry for von Willebrand factor (vWF) strongly labels dysplastic megakaryocytes, accentuating their abnormal clustering and nuclear morphology.

Workup: BM was markedly hypercellular (∼90%) with 9% eosinophils, myelofibrosis increased (MF 1-2), and frequent small, hypolobated megakaryocytes. Storage iron could not be assessed due to a lack of marrow particles on iron stain (Figure 6). Karyotype and standard FISH (*PDGFRA/B*, *BCR::ABL1*) were normal. Initial impression was myelodysplastic/myeloproliferative neoplasm unclassifiable vs reactive process.

Molecular Findings: Retrospective myeloid NGS identified pathogenic variants in *SF3B1* (45% VAF), *CBL* (48% VAF), and *BCOR* (3% VAF).

Diagnosis: Myelodysplastic/myeloproliferative neoplasm with *SF3B1* mutation and thrombocytosis.

Diagnostic Lesson: This case exemplifies how myeloid NGS can definitively reclassify “unclassifiable” eosinophilic presentations as specific clonal myeloid neoplasms under current WHO and ICC frameworks.

## DISCUSSION

In this cohort of patients with persistent, unexplained HE at the time of BM biopsy, integrated clinicopathologic evaluation frequently resolved the provisional HEPU designation. Among patients with HEPU, most cases were ultimately classified into the idiopathic spectrum (36.8%) or as reactive eosinophilia (28.9%), while a clinically important subset was reassigned to clonal disorders, including both myeloid and lymphoid neoplasms (28.9%), with a small fraction remaining in mixed reactive vs idiopathic categories (5.3%). These findings underscore that “unexplained” HE at biopsy is often a transitional label that can be clarified with structured marrow-based workup and follow-up.

A central implication of our results is the continued primacy of marrow morphology for classification. Prior work has shown that BM morphologic abnormalities are a strong discriminator between CEL NOS and reactive/idiopathic HE/HES,^8^ and this principle is reinforced in the ICC 2022 approach, which explicitly elevates morphologic criteria in the CEL NOS vs idiopathic HE/HES distinction.^34^ Our reclassifications were most often driven by morphologic evidence of myeloproliferation and/or dysplasia, interpreted in conjunction with cytopenias, fibrosis, and longitudinal course, consistent with ICC and WHO-HAEM5 clinicopathologic integration.^9^^,^^10^

Ancillary studies provided complementary value. Cytogenetics/FISH were infrequently diagnostic overall, but they remain essential to identify defining and actionable tyrosine kinase fusion–associated neoplasms.^3^^,^^9^ In our cohort, targeted testing detected *FIP1L1::PDGFRA* in 2 HEPU cases, illustrating that even rare fusion-positive diagnoses can have immediate therapeutic implications.^1^^,^^3^

Myeloid NGS contributed additional resolution (reportable variants in 47% of tested patients with HEPU) but also highlighted the risk of overinterpreting somatic variants as evidence of eosinophil-lineage neoplasia. Clonal hematopoiesis of indeterminate potential is common, increases with age, and is associated with adverse outcomes and future hematologic malignancy risk^12‐14^; accordingly, variant classification and clinical interpretation should follow established recommendations and be anchored to marrow morphology and other supportive features.^16^ Prior targeted sequencing studies similarly identify subsets of “idiopathic” HE/HES with myeloid-associated variants and features more consistent with CEL NOS, supporting selective NGS use in persistently unexplained cases.^17^ Overall, our findings support a pragmatic workflow aligned with WHO-HAEM5 and ICC 2022: exclude secondary causes, assess organ involvement, perform thorough marrow examination with cytogenetics/FISH for definitional lesions, and apply broad myeloid NGS selectively when morphology or persistent clinical course raises concern for clonality.^3^^,^^4^^,^^9^

## CONCLUSION

Our findings challenge the traditional reliance on “idiopathic” categorization for patients with persistent HE. We demonstrate that “unexplained” eosinophilia is frequently a provisional state; by applying an integrated assessment of BM morphology and myeloid NGS, we successfully reclassified nearly two-thirds of such patients into specific reactive or clonal diagnostic categories.

Targeted NGS proved indispensable in this workflow, identifying clonal variants in genes such as *SF3B1*, *CBL*, and *TP53* in nearly half of the tested cases. However, the high sensitivity of NGS introduces a new diagnostic challenge: distinguishing true CEL from background CHIP. Our data affirm that molecular clonality cannot be interpreted in isolation; it must be rigorously contextualized by BM morphology, specifically megakaryocytic dysplasia and fibrosis, to satisfy the strict criteria of the fifth edition WHO classification.

We advocate that BM evaluation with integrated NGS should be positioned as an essential early investigation for persistent unexplained HE, rather than being reserved for cases that are refractory to empiric therapy or remain undefined after exhaustive noninvasive testing.

## Data Availability

The data underlying this article will be shared on reasonable request to the corresponding author.
